# SARS-CoV-2 hot-spot mutations are significantly enriched within inverted repeats and CpG island loci

**DOI:** 10.1093/bib/bbaa385

**Published:** 2020-12-21

**Authors:** Pratik Goswami, Martin Bartas, Matej Lexa, Natália Bohálová, Adriana Volná, Jiří Červeň, Veronika Červeňová, Petr Pečinka, Vladimír Špunda, Miroslav Fojta, Václav Brázda

**Affiliations:** Department of Biophysical Chemistry and Molecular Oncology, Institute of Biophysics of the Czech Academy of Sciences, Brno, Czech Republic; National Centre for Biomolecular Research, Faculty of Science, Masaryk University, Brno, Czech Republic; Department of Biology and Ecology, Faculty of Science, University of Ostrava, Ostrava, Czech Republic; Faculty of Informatics, Masaryk University, Brno, Czech Republic; Department of Biophysical Chemistry and Molecular Oncology, Institute of Biophysics of the Czech Academy of Sciences, Brno, Czech Republic; Department of Experimental Biology, Faculty of Science, Masaryk University, Brno, Czech Republic; Department of Physics, Faculty of Science, University of Ostrava, Ostrava, Czech Republic; Department of Biology and Ecology, Faculty of Science, University of Ostrava, Ostrava, Czech Republic; Department of Mathematics, Faculty of Science, University of Ostrava, Ostrava, Czech Republic; Department of Biology and Ecology, Faculty of Science, University of Ostrava, Ostrava, Czech Republic; Department of Physics, Faculty of Science, University of Ostrava, Ostrava, Czech Republic; Global Change Research Institute of the Czech Academy of Sciences, Brno, Czech Republic; Department of Biophysical Chemistry and Molecular Oncology, Institute of Biophysics of the Czech Academy of Sciences, Brno, Czech Republic; Department of Biophysical Chemistry and Molecular Oncology, Institute of Biophysics of the Czech Academy of Sciences, Brno, Czech Republic

**Keywords:** SARS-CoV-2, inverted repeats, CpG methylation, hot spot

## Abstract

SARS-CoV-2 is an intensively investigated virus from the order *Nidovirales* (*Coronaviridae* family) that causes COVID-19 disease in humans. Through enormous scientific effort, thousands of viral strains have been sequenced to date, thereby creating a strong background for deep bioinformatics studies of the SARS-CoV-2 genome. In this study, we inspected high-frequency mutations of SARS-CoV-2 and carried out systematic analyses of their overlay with inverted repeat (IR) loci and CpG islands. The main conclusion of our study is that SARS-CoV-2 hot-spot mutations are significantly enriched within both IRs and CpG island loci. This points to their role in genomic instability and may predict further mutational drive of the SARS-CoV-2 genome. Moreover, CpG islands are strongly enriched upstream from viral ORFs and thus could play important roles in transcription and the viral life cycle. We hypothesize that hypermethylation of these loci will decrease the transcription of viral ORFs and could therefore limit the progression of the disease.

## Introduction

Due to the ongoing coronavirus pandemic, the novel severe acute respiratory syndrome coronavirus 2 (SARS-CoV-2) is a subject of emerging contemporary medical and virology research. Since the first reported case of the SARS-CoV-2-related atypical pneumonia coronavirus disease 19 (COVID-19) in December 2019, several important questions have arisen concerning the virus’s origin, phylogenesis, and therapeutic targeting [1, 2]. To date, more than 15 000 sequences of SARS-CoV-2 have been made available in the Global Initiative on Sharing All Influenza Data (GISAID) database and more than 900 sequences in the NCBI database [3]. Although the origin of this single-stranded, positive-polarity RNA virus remains unclear, several scenarios have already been suggested [4–6]. SARS-CoV-2 is likely to have evolved in bats, as 96% of its genomic sequence is identical to that of the bat coronavirus strain RaTG13 [7]. Malaysian pangolin (*Manis javanica*) has been proposed as intermediate host because Pangolin-CoV, with 91% sequence homology, is the second-closest relative of SARS-CoV-2 [8]. At the whole-genome level, SARS-CoV-2 is 82% identical to SARS-CoV [9], whereas the receptor-binding domains (RBDs) of the spike glycoprotein from the two viruses share 72% identity in amino acid sequence and similar ternary structures, but a stronger interaction of SARS-CoV-2 RBD with entry receptor angiotensin converting enzyme 2 (ACE2) has been reported [10]. Based on ACE2 sequence alignment, the potential host range was broadened to dog, cat, pangolin, and small mammals of the *Cricetidae* family [11]. For binding to human ACE2, relevance of the Q493 and P499 virus amino acid residues (corresponding to nucleotide loci 23 039–23 041 and 23 057–23 059 of the reference genome NC_045512.2) has been demonstrated, whereas the N493Q mutation from SARS-CoV-2 to SARS-CoV increased affinity to ACE2 and T499P mutation is responsible for stabilizing the interface of RBD interacting with ACE2 [12].

SARS-CoV-2 encodes for 10 canonical ORFs, including four major structural proteins: spike glycoprotein (S), membrane protein (M), envelope protein (E), and highly immunogenic and abundantly expressed nucleocapsid protein (N) [13, 14]. In addition, SARS-CoV-2 transcriptome analyses have revealed also unknown ORFs emergent by fusion, deletion, and frameshift [15]. In general, RNA viruses are characterized by high mutation rate, which enables them to evolve rapidly. Analyses of 4254 SARS-CoV-2 sequences have revealed that mutations are most commonly found inside ORF1a, ORF1b, as well as S and N genes, in contrast to ORF7b and E gene, which exhibited low frequency of mutation rate [16, 17]. Although mutations are geographically distributed, it is surprisingly the case that mutations in positions 2891, 3036, 14 408, 23 403, and 28 881 are predominantly observed in Europe, while those located at positions 17 746, 17 857, and 18 060 are mainly present in North America [18].

Several noncanonical nucleic acid structures, such as G-quadruplexes, cruciforms, hairpins, and triplexes, have been shown to be essential for genome regulation and could be the sources of genetic instability [19–22]. Although only a few G-quadruplex-forming sequences have been determined in the SARS-CoV-2 genome, its genome is abundant (in comparison with other viruses of the Nidovirales group and compared to G-quadruplex-forming sequences) in the presence of inverted repeats (IRs) [23]. IRs are nonrandomly distributed in the genomes of all living organisms and can adopt a hairpin stem-loop secondary structure in single-stranded or a cruciform structure within double-stranded nucleic acid [24, 25]. They play an important role in regulating basic biological processes in both DNA and RNA genomes and are targets for many regulatory proteins [19, 26, 27]. Recently, it was demonstrated that two conserved regions of SARS-CoV-2 and SARS-Co-V form stem-loop structures and can protect viral RNA from rapid degradation in human cell lines, thereby possibly enhancing the stability of viral RNA genomes and augmenting viral replication efficiency and virulence [28]. Moreover, the stem integrity of a phylogenetically conserved stem-loop structure located in the 5′ UTR of the PRRSV virus from the *Arteriviridae* family was confirmed to be crucial for replication and subgenomic mRNA synthesis. Similar secondary structures have been proposed that occur in several viruses among the *Arteriviridae* and *Coronaviridae* families, to which SARS-CoV-2 belongs [27]. DNA IRs have proven to be hot spots for genetic instability, with higher probability of mutations in repeats that can form secondary structures [29].

Many RNA viruses, including SARS-CoV-2, exhibit the depletion of CpG dinucleotides [30]. Two main theories have been put forward to explain CpG depletion. One is based on mutation susceptibility, as cytosine methylation increases mutational rate by spontaneous deamination of 5-methylcytosine to thymine [31]. This mutational rate has been shown to be higher when CpG is flanked by other cytosines or guanines than when flanked by thymines or adenines [32]. The other hypothesis focuses on interaction with host immune systems, as viruses are trying to match host CpG frequencies and methylation patterns. The CpG frequency in influenza virus has been shown to drop rapidly after transferring from avian to human [33]. In vertebrates- and especially in human-infecting viruses, the CpG frequency is extremely low [31]. Higher frequency of CpG has been associated with attenuation of the virus [34, 35].

Because both IRs and depletion of CpG islands influence viral replication and virulence, we decided to investigate SARS-CoV2 IR and CpG island locations in connection with their mutation potential and genome localization. We conducted a systematic and comprehensive bioinformatic study searching for the occurrence of IRs and CpG islands in relation to hot-spot mutations within the SARS-CoV-2 genome.

## Results

**Figure 1:**
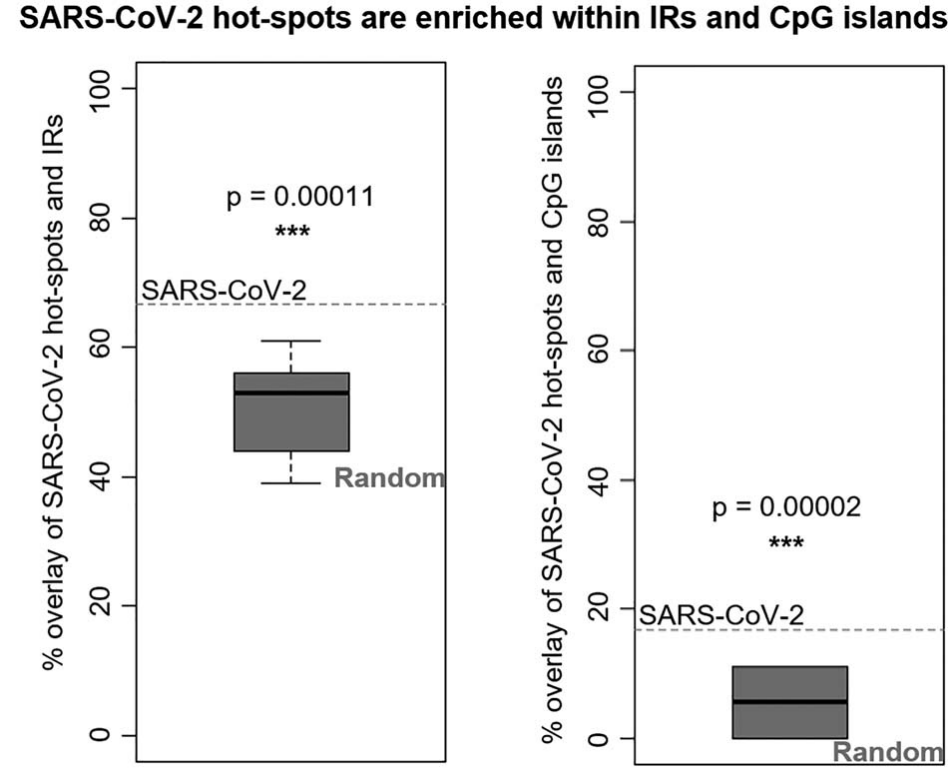
Overlay of SARS-CoV-2 hot-spot mutations with IRs in SARS-CoV-2 genome (left) and with CpG islands in SARS-CoV-2 (right) and comparison with random mutations (boxplots). One-sample *t*-test was used. ^*^^*^^*^ indicates *P*-value < 0.001.

We analyzed the presence of IRs in the whole genome of SARS-CoV-2 and created an overlay with 18 high-frequency nucleotide positions identified as hot spots based on their GISAID frequency. Among the whole set of 18 hot-spot mutations (Table 1, complete analyses in Supplementary Materials 1 and 2, available online at https://academic.oup.com/bib), 12 of them (i.e. 66.7%) lie inside IR sequences. By comparison, a set of 18 randomly placed positions (in 10 replicates) revealed a mean overlay of 50.6% with a standard deviation of 8.1%. Thus, the hot-spot mutations in SARS-CoV-2 were enriched within IRs loci and this association was highly significant statistically (*P*-value = 0.0001085; *t* = −5.94, df = 9, one-sample *t*-test) (Figure 1).

**Table 1:** SARS-CoV-2 hot-spot positions (GISAID frequency > 0.04)

RefP	RefN	AltN	FreqGis	Feature	Gene product	AltAA	Mutation*	IR	CpG
241	C	T	0.69	5′ UTR	–	–	–	N	**Y**
1059	C	T	0.21	ORF1ab	nsp2	T85I	NS	**Y**	N
1605	A	C	0.04	ORF1ab	non-structural polyprotein 1AB	N267T	NS	**Y**	N
2891	G	R	0.06	ORF1ab	nsp3	A58T	NS	**Y**	N
3037	C	T	0.65	ORF1ab	nsp3	F106F	S	N	N
8782	C	T	0.14	ORF1ab	nsp4	S76S	S	N	N
11 083	G	T	0.15	ORF1ab	nsp6	L37F	NS	**Y**	N
14 408	C	T	0.64	ORF1ab	RNA-dependent rna polymerase (nsp12)	P314L	NS	N	N
14 805	C	T	0.11	ORF1ab	RNA-dependent rna polymerase (nsp12)	Y446Y	S	**Y**	N
17 247	T	C	0.04	ORF1ab	helicase (nsp13)	R337R	S	**Y**	**Y**
17 747	C	T	0.09	ORF1ab	helicase (nsp13)	P504L	NS	**Y**	N
17 858	A	G	0.08	ORF1ab	helicase (nsp13)	Y541C	NS	**Y**	N
18 060	C	T	0.1	ORF1ab	3′-5′ exonuclease activity	L7L	S	**Y**	N
23 403	A	G	0.64	S	spike glycoprotein	D614G	NS	**Y**	N
25 563	G	T	0.24	ORF3a	ORF3a protein	Q57H	NS	N	N
26 144	G	T	0.11	OR3a	ORF3a protein	G251V	NS	N	**Y**
28 144	T	C	0.13	ORF8	ORF8 protein	L84S	NS	**Y**	N
28 881	G	A	0.2	ORF9/N	nucleocapsid phosphoprotein	R203K	NS	**Y**	N

Notes: RefP—reference position in NC_045512.2 genome, RefN—reference nucleotide, AltN—mutation according to GISAID, standard IUPAC code used, FreqGis—mutation frequency according to GISAID, Feature—annotated features according to NCBI, AltAA—mutation of amino acids, Mutation—type of mutations, IR—presence of IR, CpG—presence of CpG island

^*^S—synonymous mutation, NS—non-synonymous mutation

In six cases, hot-spot mutations were located within the stem region of an IR (nucleotide positions 1059, 2891, 17 747, 17 858, 18 060, and 28 144). In five cases, the hot-spot mutations were located within loop regions of IRs (nucleotide positions 1605, 11 083, 14 805, 17 247, and 23 403). In a single case of nucleotide position 28 881, the hot-spot mutation was located in an IR where both stem and loop could be present (overlay of two IRs, graphically shown in Supplementary Material 3). Additionally, we compared the presence of hot-spot mutations according to the length of one repeat of IR (Table 2). Those IRs with shortest lengths were most abundant in the SARS-CoV-2 genome. All hot-spot mutations resided in IRs with length up to 9 (for one repeat of the IR; the total length of the IR is then 18 for cases without spacer). Two hot-spot mutations were present in more than one IR. Mutation at position 14 805 resides in stem of IR length categories 6 and 9. Mutation at position 28 881 was present in the genome with three various IR length categories of 6, 7, and 8. Eight hot-spot mutations (53.3%) were located inside the most abundant IRs in length category 6. Longer IRs were rare, and no hot-spot mutation was observed in the IRs of length categories 10–13.

**Table 2:** Numbers and frequencies of IRs according to IR category (based on the length of one IR repeat)

IR category	Cases	IR per 1000 nt	Hot-spot mutations	% of Hot-spot mutations
6	737	24.65	1059, 1605, 2891, 11 083, 14 805, 17 747, 23 403, 28 881	53.3%
7	263	8.80	17 247, 18 060, 28 144, 28 881	26.7%
8	127	4.25	28 881	6.7%
9	39	1.30	14 805, 17 858	13.3%
10	28	0.94	NIL	NIL
11	4	0.13	NIL	NIL
12	3	0.10	NIL	NIL
13	2	0.07	NIL	NIL

Notes: Cases—Number of IRs in SARS-Cov2 genome, IR per 1000 nt—frequency of IRs per 1000 nt, Hot-spot mutations—Reference position of hot-spot mutation in NC_045512.2 genome, % of Hot-spot mutations—Hot-spot mutations percentage distributed in IRs of different sizes

Furthermore, we analyzed the presence of CpG islands in the SARS-CoV-2 genome. We found 50 CpG islands with the minimum threshold score of 17 and the maximum score of 107. The mean CpG island length was 27 nucleotides, minimum length was 3 nucleotides, and maximum length was 217 nucleotides. An overlay of CpG islands with 18 high-frequency hot-spot mutations showed that 3 hot-spot mutations (i.e. 16.7%) were located inside CpG islands. By comparison, a set of 18 randomly placed positions (in 10 replicates) revealed a mean CpG overlay of 5.0% (with a standard deviation of 4.6%). The hot-spot mutations in SARS-CoV-2 were thus enriched within CpG islands, and this association was statistically significant (*P*-value = 0.0000169; *t* = −7.58, df = 9, one-sample *t*-test) (Figure 1 and see Supplementary Material 4 available online at https://academic.oup.com/bib).

**Figure 2:**
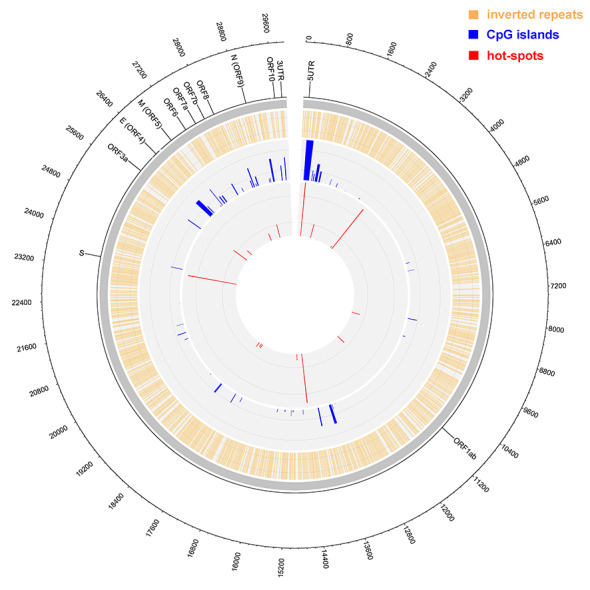
Circos plot of IRs and CpGs overlay with SARS-CoV-2 hot-spot mutations. Outer circle—nucleotide positions, second circle—gene annotations (ORFs are designated by their common symbols [S for spike glycoprotein, E for envelope protein, M for membrane glycoprotein, and N for nucleocapsid phosphoprotein]). Orange—IR presence, blue—CpG island presence (heights of CpG peaks correspond proportionally to their score by newcpgpeak [higher peak = higher score]). Red—hot-spot mutations (heights of hot-spot mutations bands proportionally express their frequencies in all analyzed genomes). The grey circle separates the descriptive (outer) and analytics (inner) part of the plot.

We constructed a Circos plot (Figure 2) to provide an overall view of the hot-spot mutations, IRs, CpG islands, and genomic features overlay. Whereas IRs occurred in ~50% of the SARS-CoV-2 genome, the CpG islands occurred mainly at the beginnings of ORFs. The most prominent CpG island was associated with the most-frequent hot-spot mutation at position 241 (5′ UTR). At the same time, the CpG island with the highest score was overlaying the transcription start site of ORF1ab, the longest transcript of SARS-CoV-2 and which encodes a polyprotein 7096 amino acid residues long. This polyprotein is subsequently cleaved by the main viral proteinase Mpro (also termed 3CLpro) to form important functional proteins [36].

To further validate our results, we have compared both CpG islands and IRs overlay with the mutation dataset published by the Balloux group. Those authors had focused on mutations that have emerged independently multiple times (i.e. homoplasies) in the SARS-CoV-2 genome [37]. In their study, they found 198 recurrent mutations that occur with various frequencies in SARS-CoV-2 sequencing data. Our analyses of IRs showed that 92 of those 198 mutations are inside IRs (46.4%) compared with 68.29 ± 5.29 (34.49%) for 100 repetitions of randomly placed mutations. Thus, the recurrent mutations in SARS-CoV-2 were enriched within IRs and this association was highly significant statistically (*P*-value < 2.2e−16; *t* = −35.55, df = 99, one-sample *t*-test). Analyses of CpG islands overlay with these mutations show that 19 of 198 mutations lie within the CpG islands (9.60%). In comparison, for 100 repetitions of random mutation placement, only 9.21 ± 2.23 (4.65%) were found in CpGs. The recurrent mutations in SARS-CoV-2 were thus enriched within CpG islands, and this association was statistically significant (*P*-value < 2.2e−16; *t* = −35.70, df = 99, one-sample *t*-test). Both comparisons confirm our results concerning hot-spot mutations, showing that mutation rates are significantly enhanced in both IRs and CpG island regions (see Supplementary Material 5 available online at https://academic.oup.com/bib).

## Discussion and conclusions

Epigenetic modifications and noncanonical nucleic acid structures play essential roles in regulating and organizing genomes [19, 36, 38]. It has been demonstrated that G-quadruplex formation regulates vital RNA syntheses [39]. In the case of the SARS-COV-2 genome, however, it was shown that potential G-quadruplex-forming sequences occur very rarely [23, 40], and thus, G-quadruplexes are probably evolutionarily eliminated. This suggestion is supported by a recent finding that SARS-COV-2 genomes exhibit an accumulation of C > U mutations and CpG depletion [6]. Therefore, we have focused on hot-spot mutations in the SARS-COV-2 genome. Our selection of hot spots is very similar to that used by the Balloux group [37], but we have applied a much stricter threshold and the more recent database set of SARS-COV-2 genomes. The majority of the hot spots from that group’s dataset are the same as we found and confirmed our results. The significant abundance of mutations in IRs and CpG islands is also valid for the dataset of all recurrent mutations found by van Dorp *et al*. [37]. It is notable that SARS-COV-2 hot-spot mutations are significantly abundant in IR sequences and CpG islands, thus suggesting the SARS-COV-2 genome’s possible survival strategy and/or evolutionary benefit to the virus in either adapting to human host, modulating cellular immune response, or even increasing virulence and pathogenicity. IRs are generally very important for ssRNA genome organization [41–43]. From 18 high-frequency hot-spot mutations, we observed 12 hot-spot mutations as nonsynonymous mutations, 5 as synonymous with no changes in protein sequence, and 1 of these hot-spot mutations being present at 5′ UTR. The majority of the mutations therefore change the protein sequence and can contribute to rapid modifications of their function and immunogenicity. Our analyses showed that CpG islands were located at the beginnings of ORFs, thus pointing to their essential regulatory roles in the SARS-CoV-2 lifecycle. On the other hand, CpG islands in RNA are very often targets of methylation enzymes and it has been demonstrated that viral genome’s methylation could lead to the inhibition of both DNA and RNA viruses [44, 45]. Interestingly, there is correlation between folate-related enzyme mutation [methylenetetrahydrofolate reductase (MTHFR)] and the COVID-19 disease’s severity. The point mutation of the MTHFR gene at position 677 causes thermolability and decreased activity of this enzyme [46, 47], and the mutated 677 allele is very common in Italy, Spain, and Hispanic populations (more than 20%) compared with other populations [47]. Notably, these same nations and groups (Italy, Spain, and Brazil) have been among those most affected by the COVID-19 pandemic. It has been demonstrated that methylation status can be significantly influenced by nutrition and folic acid supplementation [48]. The importance of micronutrients and folate in viral methylation status regulation is supported by several papers. For example, it has been shown that folate plays an important role in maintaining high methylation status at CpG sites of the human papillomavirus (HPV) and is associated with decreased risk in HPV-associated cervical intraepithelial neoplasia [49, 50]. Several polymorphisms in the folate-metabolizing MTHFR enzyme are associated with hypertensive patients’ response to riboflavin supplementation [51].

**Figure 3:**
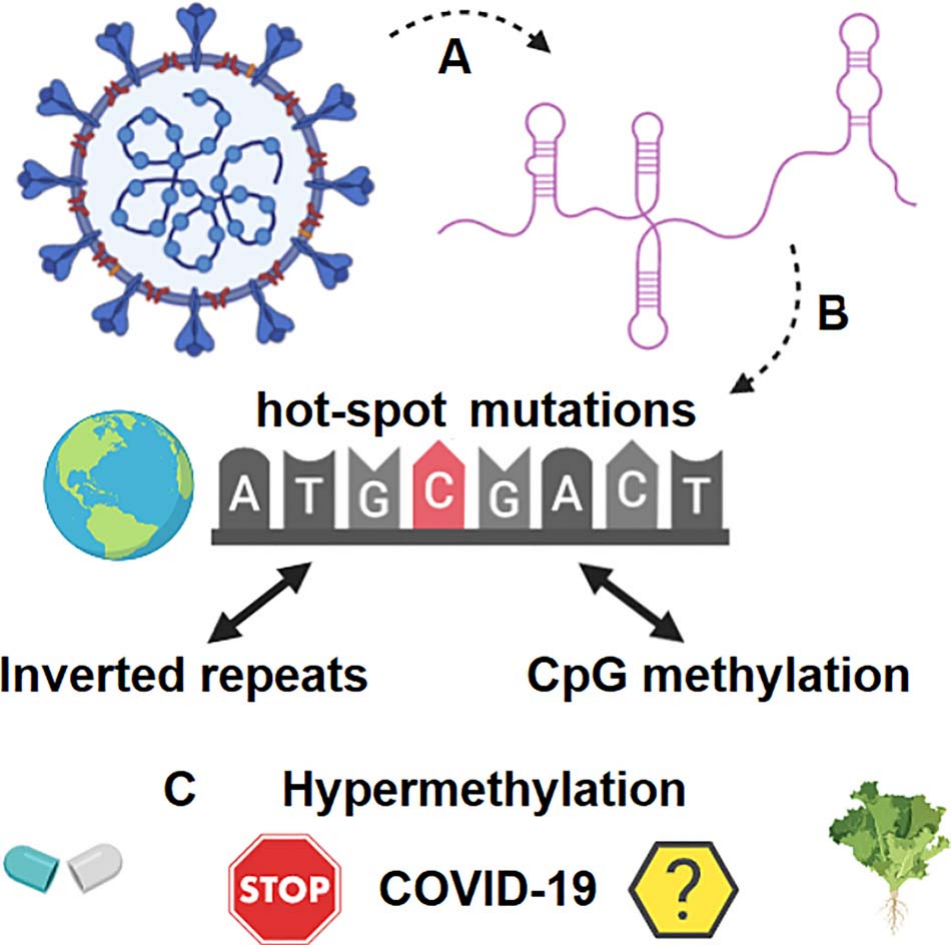
Scheme of knowledge and hypotheses proposed from the IR and CpG island overlay with SARS-CoV-2 hot-spot mutations. The SARS-CoV-2 RNA genome is organized by IRs (**A**) [23], which are significantly enriched in hot-spot mutations together with CpG islands (**B**). Hypermethylation of CpG islands could be a promising strategy for decreasing activity of the virus (**C**).

It has been shown that epigenetic modifications as well as local nucleic acid structures are possible therapeutic targets in viral genomes [52, 53]. Both G-quadruplexes and hairpins formed by IRs are recognized by many cellular proteins [19, 26, 54]. Although IRs are essential for viral genome organization, it seems that G-quadruplexes have been effectively eliminated in order to help the virus to circumvent cellular immunity [23, 55, 56]. Nevertheless, the association of hot-spot mutations with IR loci suggests also selective pressure against hairpins at specific locations. Abundance of mutations in CpG islands in the SARS-CoV-2 genome points to CpG methylation’s importance. CpG islands in human viruses have been shown to be targeted by several proteins that are part of the anti-viral defence system. In HIV virus, for example, an increase of CpG methylation led to a decrease in virulence [32]. Our data lead us to hypothesize that the hypermethylation of CpG islands could lead to the reduced transcription of SARS-CoV-2 ORFs and limit disease progression (Figure 3) and that folate supplementation could be beneficial and decrease the risks associated with SARS-CoV-2 infection.

## Materials and methods

### Hot-spot mutations selection

Single nucleotide polymorphisms in SARS-CoV-2 sequences were searched using snp-sites software [57] and the -v switch to produce VCF files. All reported differences were summed and the total divided by the total number of sequences (15 290 as of 5 May 2020 in GISAID data; 942 as of 23 April 2020 in NCBI data). Positions in regions with high proportions of Ns and ‘-’ symbols (reference genome coordinates 1–47 or 29 834–29 903) were ignored. After removing these, the remaining VCF columns of the filtered file were used for further analysis. The GISAID analysis used multiple sequence alignment file (msa_0506.fasta downloaded 6 May 2020) as input for snp-sites. For the NCBI data, the sequences were aligned to the reference sequence using blastn [58] with -outfmt 0, which was then converted to multiFASTA alignment using mview [59]. From this SNP analysis outcome, we chose only those hot-spot positions with GISAID frequency >0.04. This cut-off’s determination was based upon the inspection of SNP frequency histogram to determine which SNP percentages lay in a long tail of the curve, representing reference genome positions that are mutated more often than a general background of random mutations and possibly even sequencing errors.

### Analyses of IRs

The SARS-CoV-2 genome (NC_045512.2) was analyzed by the core of the Palindrome analyzer webserver [60]. The size of one repeat unit of IRs was set to 6–30 nt, size of spacers to 0–10 nt, and a maximum 1 mismatch was allowed. The IR has been categorized according to the length of one repeat (e.g. the length of IR in category ‘6’ without spacer is therefore 12 nt). The overlay of IRs with hot-spot mutations and randomly generated mutations is presented in Supplementary Material 1 available online at https://academic.oup.com/bib. Overlay of hot-spot mutations with IRs of individual length categories is presented in Supplementary Material 2 available online at https://academic.oup.com/bib.

### CpG islands determination

A reference sequence of a SARS-CoV-2 complete genome (NC_045512.2) was downloaded from the NCBI database in FASTA format and uploaded into the GALAXY web server [61]. The newcpgseek tool [62] with threshold 17 was used for determining CpG islands in SARS-CoV-2. Similarly, we processed the reverse complement sequence, which was derived from the SARS-CoV-2 complete genome [63], then uploaded into the GALAXY web server and also processed using the newcpgseek tool. The detailed output is provided in Supplementary Material 4 available online at https://academic.oup.com/bib. The prediction program newcpgseek uses a running sum to produce a score: if there is not a CpG at position *i*, then decrement runSum counter, but if CpG then runSum+ (=CPG SCORE). Spans above the threshold are searched for recursively. If the score is higher than a threshold, then a putative island is declared.

### Statistical analyses

One-sample *t*-test was used for the statistical comparison of SARS-CoV-2 hot-spot mutations and randomly placed hot-spot overlays (10 replicates) with the SARS-CoV-2 IRs and CpGs. A standard *P*-value threshold (0.05) was applied.

**Key Points**

- SARS-CoV-2 hot-spot mutations are localized nonrandomly in its genome.
- Hot-spot mutations are significantly enriched within inverted repeats and CpG island loci.
- CpG islands are also associated with upstream regions of viral ORFs.

## Supplementary Material

Sm_1_bbaa385

sm_2_bbaa385

SM_03_bbaa385

SM_04_bbaa385

SM_05_bbaa385

## Data Availability

All data are avaiable in the paper and in the Supplementary data.

## Notes

**Pratik Goswami** is carrying out his PhD work at the Institute of Biophysics of the Czech Academy of Sciences, Brno, Czech Republic. He is a student of the Faculty of Science, Masaryk University, Brno, Czech Republic. His research focus includes study of nucleic acids structures and their interactions with proteins.

**Martin Bartas** is a postdoc in the Department of Biology, University of Ostrava, Czech Republic. His research interests include noncanonical forms of nucleic acids, protein interactions, and bioinformatics.

**Matej Lexa** is a Researcher at the Faculty of Informatics, Masaryk University, Brno, Czech Republic. His research focus includes plant biology, bioinformatics, and transposable elements.

**Natália Bohálová** is a PhD student at the Institute of Biophysics of the Czech Academy of Sciences, Brno, Czech Republic. Her research interests include protein–DNA interactions, molecular immunology, virology, and bioinformatics.

**Adriana Volná** is a PhD student in the Department of Physics, University of Ostrava, Czech Republic. Her work spans molecular virology, plant biology, and interdisciplinary approaches.

**Jiří Červeň** works as a research fellow in the Department of Biology, University of Ostrava, Czech Republic. His work spans molecular biology and microbiology.

**Veronika Červeňová** is a PhD student in the Department of Mathematics. Her main focus is generalized fuzzy syllogisms and their application on natural data, with particular interest in algorithmicizing.

**Petr Pečinka** is an Assistant Professor and the Team Leader of the Molecular Biology group in the Department of Biology, University of Ostrava, Czech Republic.

**Vladimír Špunda** is an Assistant Professor and the Head of the Department of Physics, University of Ostrava, Czech Republic. Vladimír does research in biophysics, biochemistry, and ecophysiology of photosynthesis.

**Miroslav Fojta** is an Assistant Professor and the Head of the Department of Biophysical Chemistry and Molecular Oncology, Institute of Biophysics of the Czech Academy of Sciences, Brno, Czech Republic. He is involved in studies of DNA structures in solution and at surfaces, DNA damage and chemical modification, and DNA-protein interactions.

**Václav Brázda** is an Assistant Professor and the Head of the Laboratory of Protein–DNA Interactions, Institute of Biophysics of the Czech Academy of Sciences, Brno, Czech Republic. He is studying the interaction of proteins with DNA, local DNA structures, and p53 protein and is a co-author of a web-bioinformatics server.
