# Supplementary material for: SARS-CoV-2 hot-spot mutations are significantly enriched within inverted repeats and CpG island loci
Source: Brief Bioinform. 2020 Dec 21;22(2):1338–45. doi: 10.1093/bib/bbaa385 (PMC7799342; doi:10.1093/bib/bbaa385)

Supplementary material 3:

A-mutation in the stem part of the hairpin
B-mutation in the loop part of the hairpin

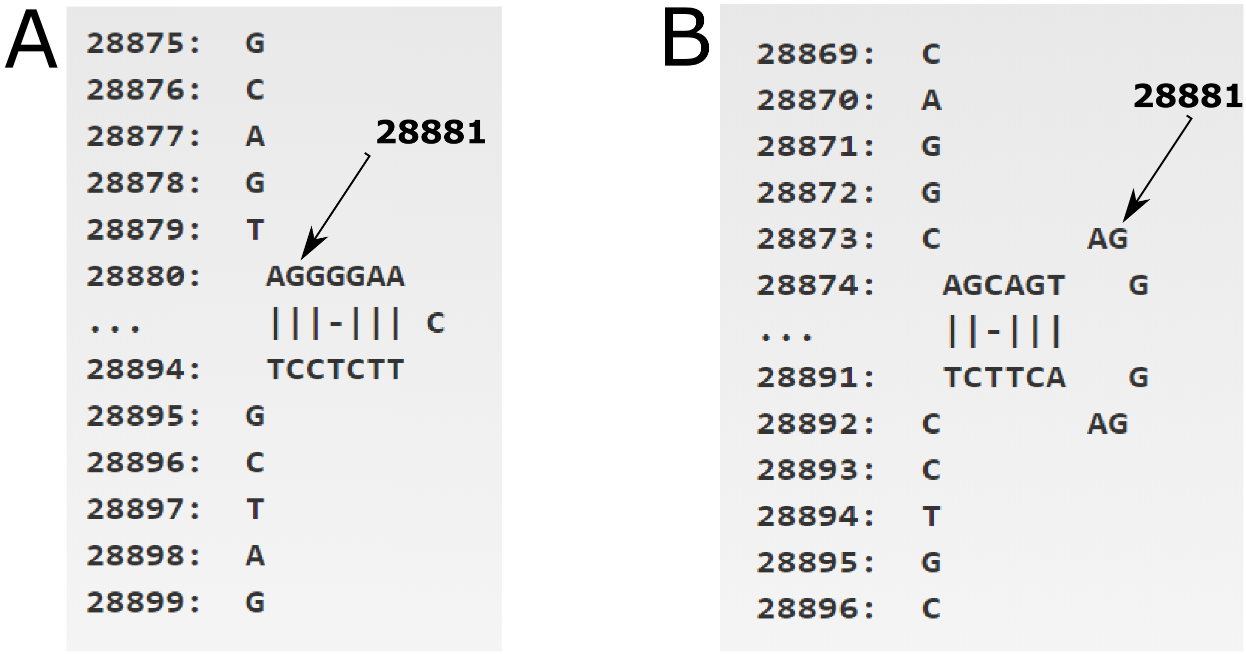

Supplement: SM_03_bbaa385 [file sm_03_bbaa385.pdf]
